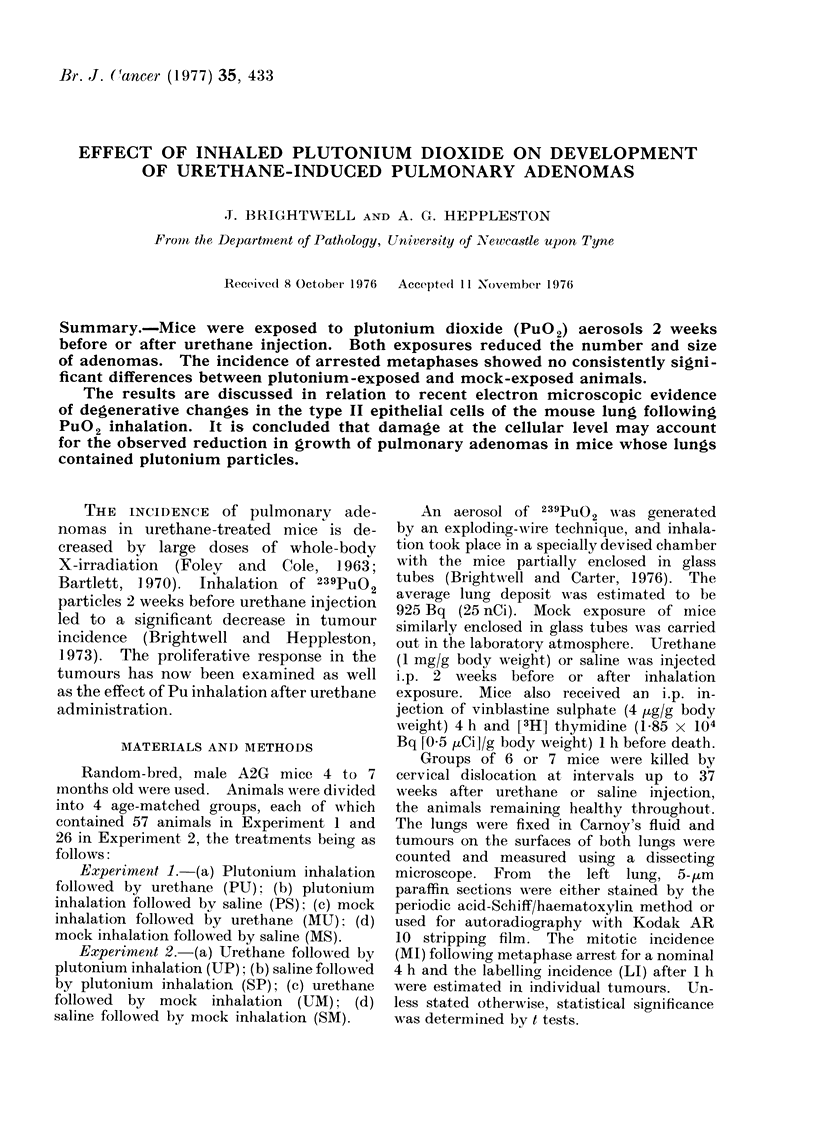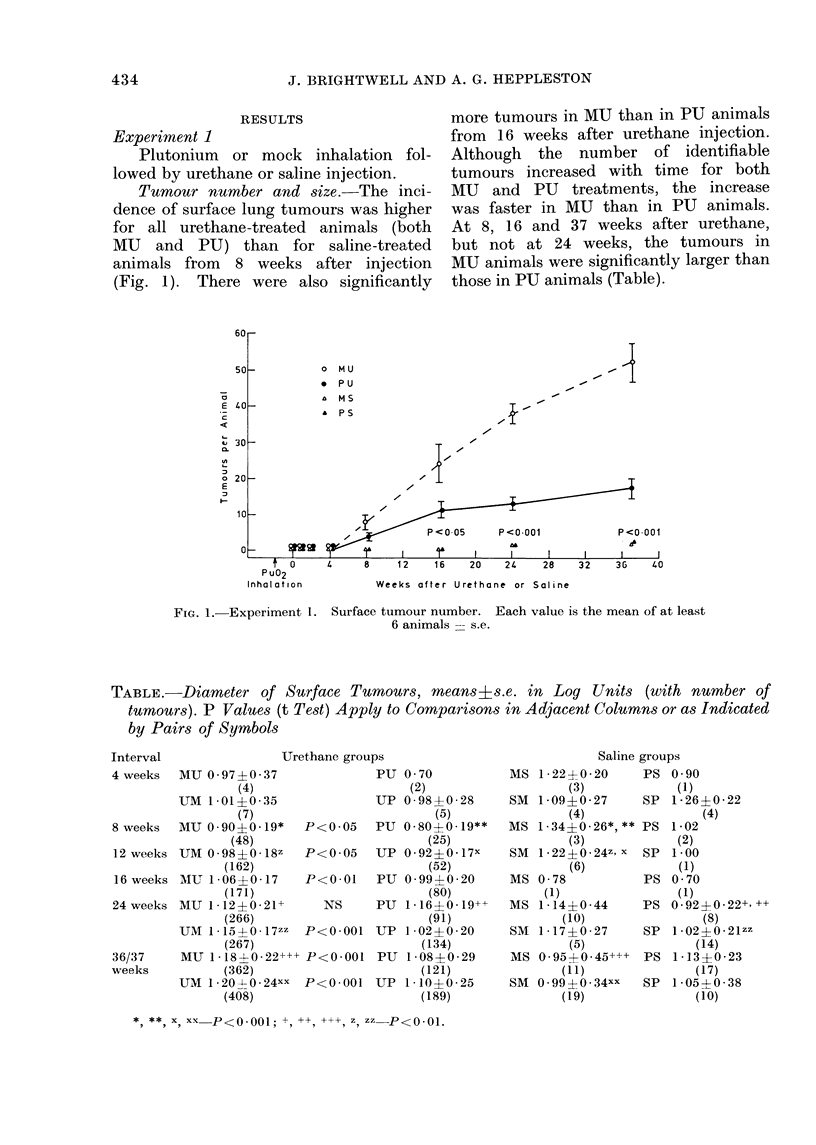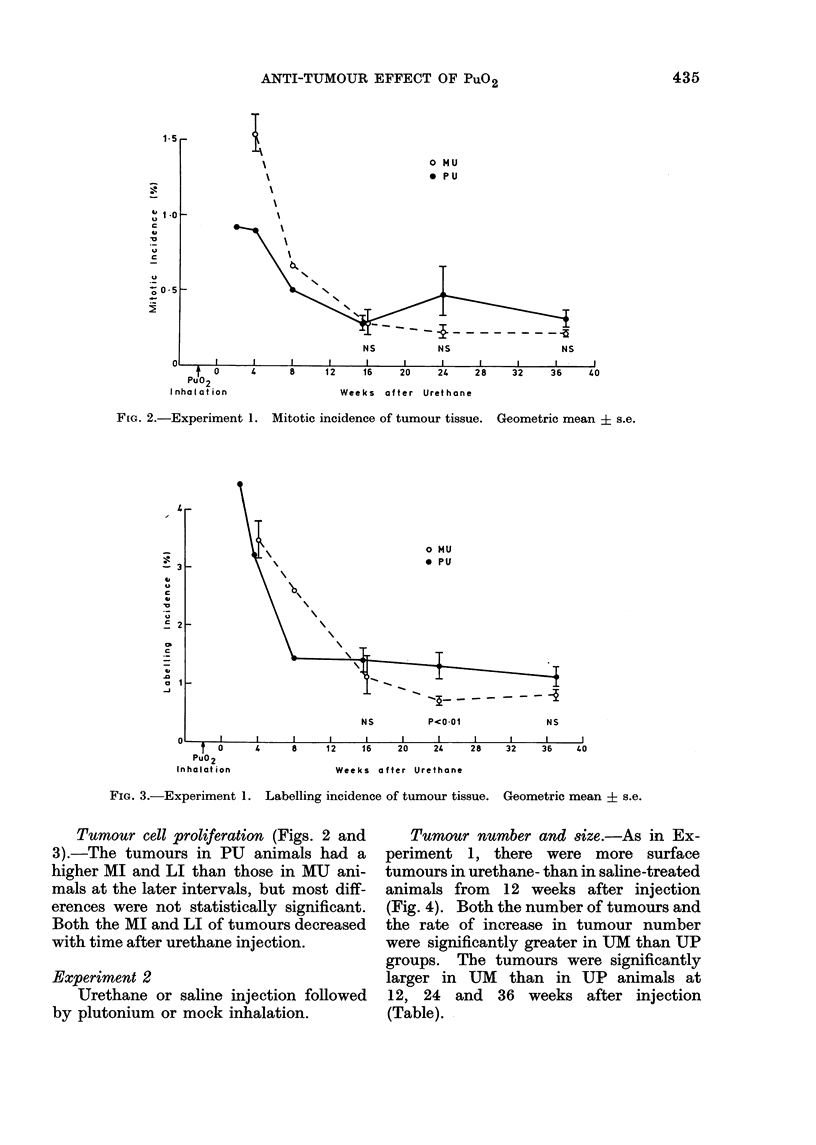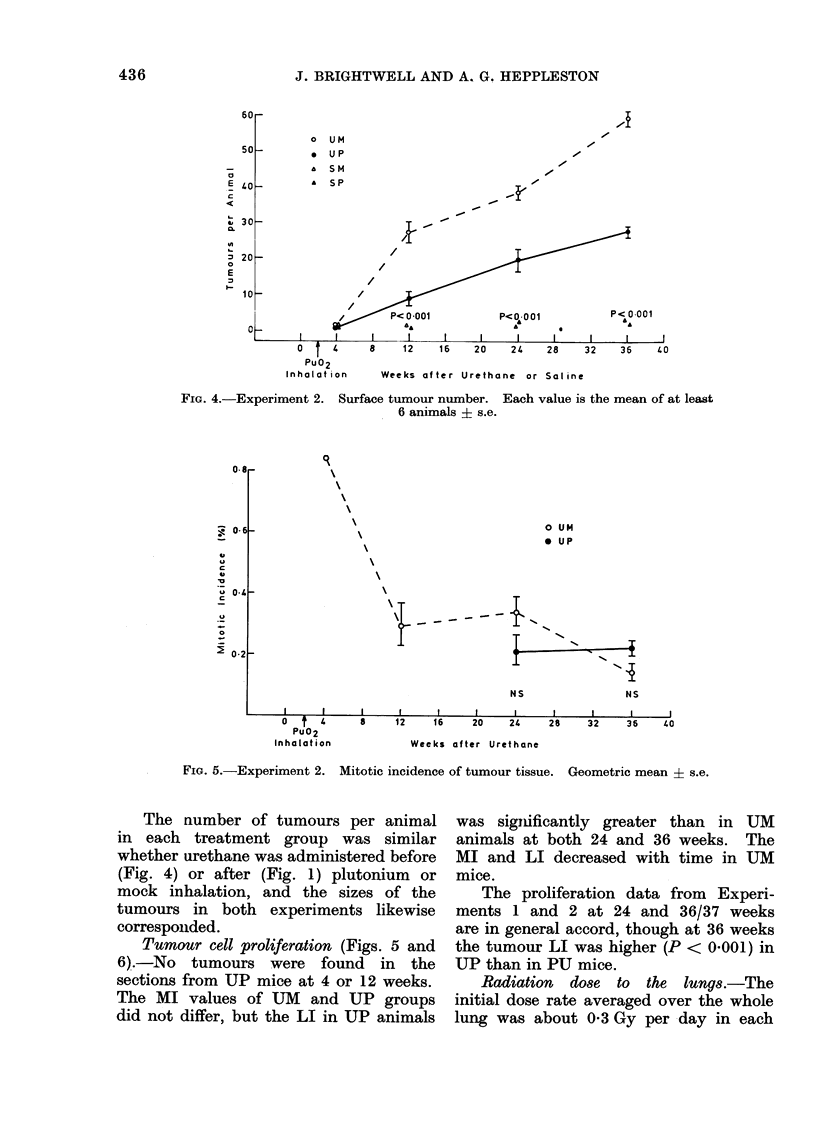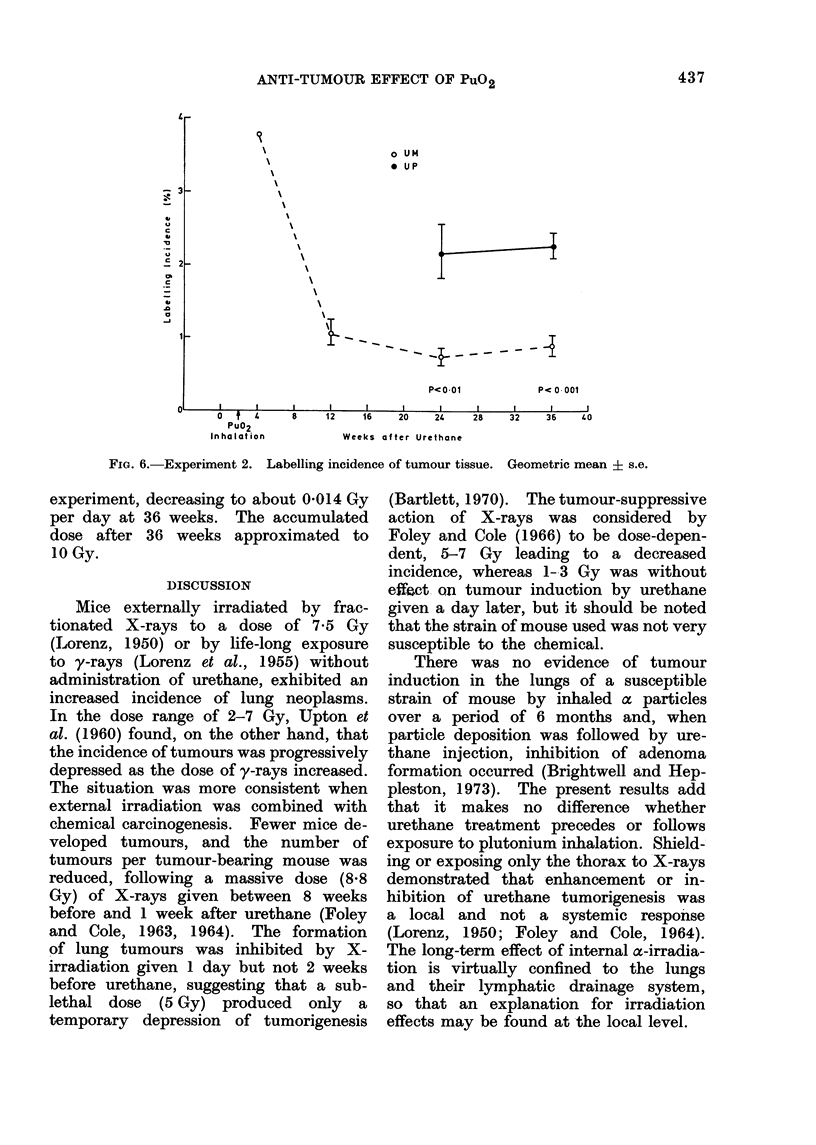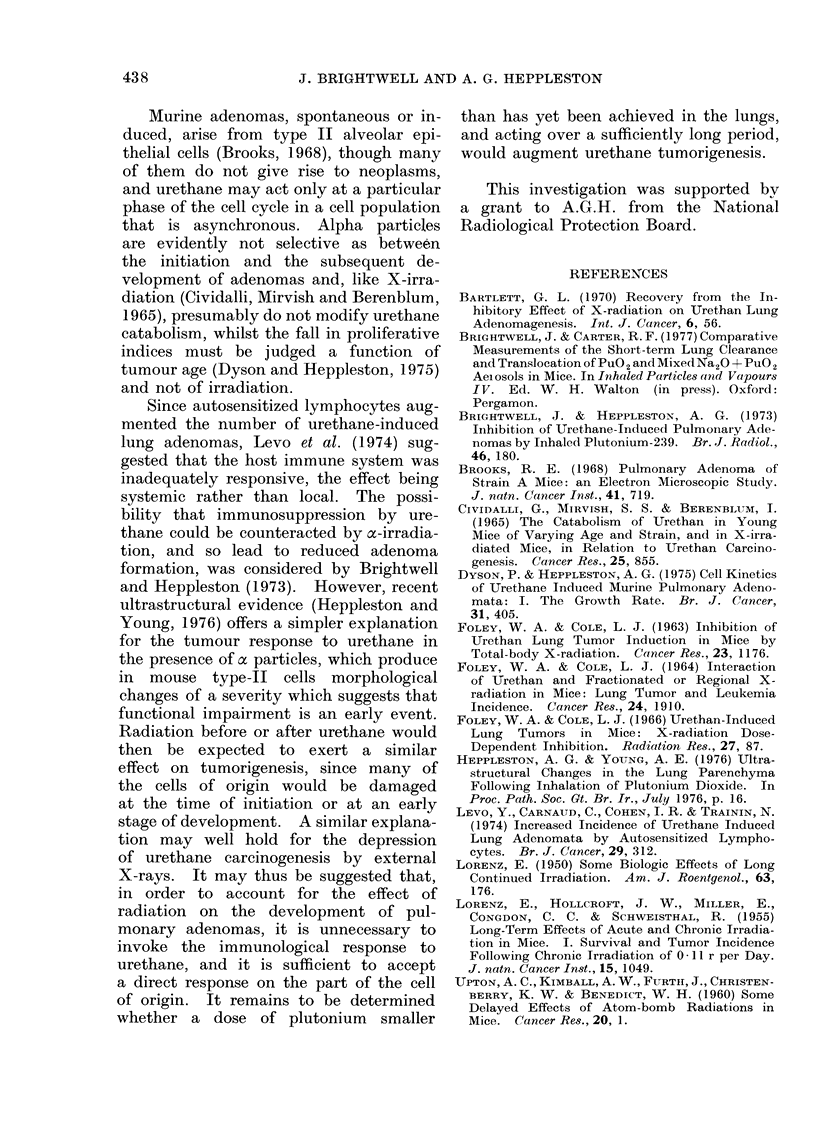# Effect of inhaled plutonium dioxide on development of urethan-induced pulmonary adenomas.

**DOI:** 10.1038/bjc.1977.65

**Published:** 1977-04

**Authors:** J. Brightwell, A. G. Heppleston

## Abstract

Mice were exposed to plutonium dioxide (PuO2) aerosols 2 weeks before or after urethan injection. Both exposures reduced the number and size of adenomas. The incidence of arrested metaphases showed no consistently significant differences between plutonium-exposed and mock-exposed animals. The results are discussed in relation to recent electron microscopic evidence of degenerative changes in the type II epithelial cells of the mouse lung following PuO2 inhalation. It is concluded that damage at the cellular level may account for the observed reduction in growth of pulmonary adenomas in mice whose lungs contained plutonium particles.


*Br. J. Cancer* (1977) **35**, 433

### EFFECT OF INHALED PLUTONIUM DIOXIDE ON DEVELOPMENT OF URETHANE-INDUCED PULMONARY ADENOMAS

J. BRIGHTWELL AND A. G. HEPPLESTON

*From the Department of Pathology, University of Newcastle upon Tyne*

Received 8 October 1976 Accepted 11 November 1976

**Summary.—Mice were exposed to plutonium dioxide ($PuO_2$) aerosols 2 weeks before or after urethane injection. Both exposures reduced the number and size of adenomas. The incidence of arrested metaphases showed no consistently significant differences between plutonium-exposed and mock-exposed animals.**

**The results are discussed in relation to recent electron microscopic evidence of degenerative changes in the type II epithelial cells of the mouse lung following $PuO_2$ inhalation. It is concluded that damage at the cellular level may account for the observed reduction in growth of pulmonary adenomas in mice whose lungs contained plutonium particles.**

THE INCIDENCE of pulmonary adenomas in urethane-treated mice is decreased by large doses of whole-body X-irradiation (Foley and Cole, 1963; Bartlett, 1970). Inhalation of $^{239}PuO_2$ particles 2 weeks before urethane injection led to a significant decrease in tumour incidence (Brightwell and Heppleston, 1973). The proliferative response in the tumours has now been examined as well as the effect of Pu inhalation after urethane administration.

### MATERIALS AND METHODS

Random-bred, male A2G mice 4 to 7 months old were used. Animals were divided into 4 age-matched groups, each of which contained 57 animals in Experiment 1 and 26 in Experiment 2, the treatments being as follows:

*Experiment 1.*—(a) Plutonium inhalation followed by urethane (PU); (b) plutonium inhalation followed by saline (PS); (c) mock inhalation followed by urethane (MU); (d) mock inhalation followed by saline (MS).

*Experiment 2.*—(a) Urethane followed by plutonium inhalation (UP); (b) saline followed by plutonium inhalation (SP); (c) urethane followed by mock inhalation (UM); (d) saline followed by mock inhalation (SM).

An aerosol of $^{239}PuO_2$ was generated by an exploding-wire technique, and inhalation took place in a specially devised chamber with the mice partially enclosed in glass tubes (Brightwell and Carter, 1976). The average lung deposit was estimated to be 925 Bq (25 nCi). Mock exposure of mice similarly enclosed in glass tubes was carried out in the laboratory atmosphere. Urethane (1 mg/g body weight) or saline was injected i.p. 2 weeks before or after inhalation exposure. Mice also received an i.p. injection of vinblastine sulphate (4 $\mu$g/g body weight) 4 h and [$^3$H] thymidine ($1{\cdot}85 \times 10^4$ Bq [0·5 $\mu$Ci]/g body weight) 1 h before death.

Groups of 6 or 7 mice were killed by cervical dislocation at intervals up to 37 weeks after urethane or saline injection, the animals remaining healthy throughout. The lungs were fixed in Carnoy's fluid and tumours on the surfaces of both lungs were counted and measured using a dissecting microscope. From the left lung, 5-$\mu$m paraffin sections were either stained by the periodic acid-Schiff/haematoxylin method or used for autoradiography with Kodak AR 10 stripping film. The mitotic incidence (MI) following metaphase arrest for a nominal 4 h and the labelling incidence (LI) after 1 h were estimated in individual tumours. Unless stated otherwise, statistical significance was determined by *t* tests.

RESULTS

*Experiment 1*

Plutonium or mock inhalation followed by urethane or saline injection.

*Tumour number and size.*—The incidence of surface lung tumours was higher for all urethane-treated animals (both MU and PU) than for saline-treated animals from 8 weeks after injection (Fig. 1). There were also significantly more tumours in MU than in PU animals from 16 weeks after urethane injection. Although the number of identifiable tumours increased with time for both MU and PU treatments, the increase was faster in MU than in PU animals. At 8, 16 and 37 weeks after urethane, but not at 24 weeks, the tumours in MU animals were significantly larger than those in PU animals (Table).

FIG. 1.—Experiment 1. Surface tumour number. Each value is the mean of at least 6 animals ± s.e.

TABLE.—*Diameter of Surface Tumours, means±s.e. in Log Units (with number of tumours). P Values (t Test) Apply to Comparisons in Adjacent Columns or as Indicated by Pairs of Symbols*

| Interval | Urethane groups | | | Saline groups | |
|---|---|---|---|---|---|
| 4 weeks | MU 0·97±0·37 (4) | | PU 0·70 (2) | MS 1·22±0·20 (3) | PS 0·90 (1) |
| | UM 1·01±0·35 (7) | | UP 0·98±0·28 (5) | SM 1·09±0·27 (4) | SP 1·26±0·22 (4) |
| 8 weeks | MU 0·90±0·19* (48) | $P<0{\cdot}05$ | PU 0·80±0·19** (25) | MS 1·34±0·26*, ** (3) | PS 1·02 (2) |
| 12 weeks | UM 0·98±0·18$^{z}$ (162) | $P<0{\cdot}05$ | UP 0·92±0·17$^{x}$ (52) | SM 1·22±0·24$^{z, x}$ (6) | SP 1·00 (1) |
| 16 weeks | MU 1·06±0·17 (171) | $P<0{\cdot}01$ | PU 0·99±0·20 (80) | MS 0·78 (1) | PS 0·70 (1) |
| 24 weeks | MU 1·12±0·21$^{+}$ (266) | NS | PU 1·16±0·19$^{++}$ (91) | MS 1·14±0·44 (10) | PS 0·92±0·22$^{+, ++}$ (8) |
| | UM 1·15±0·17$^{zz}$ (267) | $P<0{\cdot}001$ | UP 1·02±0·20 (134) | SM 1·17±0·27 (5) | SP 1·02±0·21$^{zz}$ (14) |
| 36/37 weeks | MU 1·18±0·22$^{+++}$ (362) | $P<0{\cdot}001$ | PU 1·08±0·29 (121) | MS 0·95±0·45$^{+++}$ (11) | PS 1·13±0·23 (17) |
| | UM 1·20±0·24$^{xx}$ (408) | $P<0{\cdot}001$ | UP 1·10±0·25 (189) | SM 0·99±0·34$^{xx}$ (19) | SP 1·05±0·38 (10) |

*, **, $^{x}$, $^{xx}$—$P<0{\cdot}001$; $^{+}$, $^{++}$, $^{+++}$, $^{z}$, $^{zz}$—$P<0{\cdot}01$.

FIG. 2.—Experiment 1. Mitotic incidence of tumour tissue. Geometric mean ± s.e.

FIG. 3.—Experiment 1. Labelling incidence of tumour tissue. Geometric mean ± s.e.

*Tumour cell proliferation* (Figs. 2 and 3).—The tumours in PU animals had a higher MI and LI than those in MU animals at the later intervals, but most differences were not statistically significant. Both the MI and LI of tumours decreased with time after urethane injection.

*Experiment 2*

Urethane or saline injection followed by plutonium or mock inhalation.

*Tumour number and size.*—As in Experiment 1, there were more surface tumours in urethane- than in saline-treated animals from 12 weeks after injection (Fig. 4). Both the number of tumours and the rate of increase in tumour number were significantly greater in UM than UP groups. The tumours were significantly larger in UM than in UP animals at 12, 24 and 36 weeks after injection (Table).

FIG. 4.—Experiment 2. Surface tumour number. Each value is the mean of at least 6 animals $\pm$ s.e.

FIG. 5.—Experiment 2. Mitotic incidence of tumour tissue. Geometric mean $\pm$ s.e.

The number of tumours per animal in each treatment group was similar whether urethane was administered before (Fig. 4) or after (Fig. 1) plutonium or mock inhalation, and the sizes of the tumours in both experiments likewise corresponded.

*Tumour cell proliferation* (Figs. 5 and 6).—No tumours were found in the sections from UP mice at 4 or 12 weeks. The MI values of UM and UP groups did not differ, but the LI in UP animals was significantly greater than in UM animals at both 24 and 36 weeks. The MI and LI decreased with time in UM mice.

The proliferation data from Experiments 1 and 2 at 24 and 36/37 weeks are in general accord, though at 36 weeks the tumour LI was higher ($P < 0{\cdot}001$) in UP than in PU mice.

*Radiation dose to the lungs*.—The initial dose rate averaged over the whole lung was about 0·3 Gy per day in each

FIG. 6.—Experiment 2. Labelling incidence of tumour tissue. Geometric mean ± s.e.

experiment, decreasing to about 0·014 Gy per day at 36 weeks. The accumulated dose after 36 weeks approximated to 10 Gy.

### DISCUSSION

Mice externally irradiated by fractionated X-rays to a dose of 7·5 Gy (Lorenz, 1950) or by life-long exposure to $\gamma$-rays (Lorenz *et al.*, 1955) without administration of urethane, exhibited an increased incidence of lung neoplasms. In the dose range of 2–7 Gy, Upton *et al.* (1960) found, on the other hand, that the incidence of tumours was progressively depressed as the dose of $\gamma$-rays increased. The situation was more consistent when external irradiation was combined with chemical carcinogenesis. Fewer mice developed tumours, and the number of tumours per tumour-bearing mouse was reduced, following a massive dose (8·8 Gy) of X-rays given between 8 weeks before and 1 week after urethane (Foley and Cole, 1963, 1964). The formation of lung tumours was inhibited by X-irradiation given 1 day but not 2 weeks before urethane, suggesting that a sublethal dose (5 Gy) produced only a temporary depression of tumorigenesis (Bartlett, 1970). The tumour-suppressive action of X-rays was considered by Foley and Cole (1966) to be dose-dependent, 5–7 Gy leading to a decreased incidence, whereas 1–3 Gy was without effect on tumour induction by urethane given a day later, but it should be noted that the strain of mouse used was not very susceptible to the chemical.

There was no evidence of tumour induction in the lungs of a susceptible strain of mouse by inhaled $\alpha$ particles over a period of 6 months and, when particle deposition was followed by urethane injection, inhibition of adenoma formation occurred (Brightwell and Heppleston, 1973). The present results add that it makes no difference whether urethane treatment precedes or follows exposure to plutonium inhalation. Shielding or exposing only the thorax to X-rays demonstrated that enhancement or inhibition of urethane tumorigenesis was a local and not a systemic response (Lorenz, 1950; Foley and Cole, 1964). The long-term effect of internal $\alpha$-irradiation is virtually confined to the lungs and their lymphatic drainage system, so that an explanation for irradiation effects may be found at the local level.

Murine adenomas, spontaneous or induced, arise from type II alveolar epithelial cells (Brooks, 1968), though many of them do not give rise to neoplasms, and urethane may act only at a particular phase of the cell cycle in a cell population that is asynchronous. Alpha particles are evidently not selective as between the initiation and the subsequent development of adenomas and, like X-irradiation (Cividalli, Mirvish and Berenblum, 1965), presumably do not modify urethane catabolism, whilst the fall in proliferative indices must be judged a function of tumour age (Dyson and Heppleston, 1975) and not of irradiation.

Since autosensitized lymphocytes augmented the number of urethane-induced lung adenomas, Levo *et al.* (1974) suggested that the host immune system was inadequately responsive, the effect being systemic rather than local. The possibility that immunosuppression by urethane could be counteracted by $\alpha$-irradiation, and so lead to reduced adenoma formation, was considered by Brightwell and Heppleston (1973). However, recent ultrastructural evidence (Heppleston and Young, 1976) offers a simpler explanation for the tumour response to urethane in the presence of $\alpha$ particles, which produce in mouse type-II cells morphological changes of a severity which suggests that functional impairment is an early event. Radiation before or after urethane would then be expected to exert a similar effect on tumorigenesis, since many of the cells of origin would be damaged at the time of initiation or at an early stage of development. A similar explanation may well hold for the depression of urethane carcinogenesis by external X-rays. It may thus be suggested that, in order to account for the effect of radiation on the development of pulmonary adenomas, it is unnecessary to invoke the immunological response to urethane, and it is sufficient to accept a direct response on the part of the cell of origin. It remains to be determined whether a dose of plutonium smaller than has yet been achieved in the lungs, and acting over a sufficiently long period, would augment urethane tumorigenesis.

This investigation was supported by a grant to A.G.H. from the National Radiological Protection Board.

### REFERENCES

BARTLETT, G. L. (1970) Recovery from the Inhibitory Effect of X-radiation on Urethan Lung Adenomagenesis. *Int. J. Cancer*, **6**, 56.

BRIGHTWELL, J. & CARTER, R. F. (1977) Comparative Measurements of the Short-term Lung Clearance and Translocation of $PuO_2$ and Mixed $Na_2O + PuO_2$ Aerosols in Mice. In *Inhaled Particles and Vapours IV*. Ed. W. H. Walton (in press). Oxford: Pergamon.

BRIGHTWELL, J. & HEPPLESTON, A. G. (1973) Inhibition of Urethane-Induced Pulmonary Adenomas by Inhaled Plutonium-239. *Br. J. Radiol.*, **46**, 180.

BROOKS, R. E. (1968) Pulmonary Adenoma of Strain A Mice: an Electron Microscopic Study. *J. natn. Cancer Inst.*, **41**, 719.

CIVIDALLI, G., MIRVISH, S. S. & BERENBLUM, I. (1965) The Catabolism of Urethan in Young Mice of Varying Age and Strain, and in X-irradiated Mice, in Relation to Urethan Carcinogenesis. *Cancer Res.*, **25**, 855.

DYSON, P. & HEPPLESTON, A. G. (1975) Cell Kinetics of Urethane Induced Murine Pulmonary Adenomata: I. The Growth Rate. *Br. J. Cancer*, **31**, 405.

FOLEY, W. A. & COLE, L. J. (1963) Inhibition of Urethan Lung Tumor Induction in Mice by Total-body X-radiation. *Cancer Res.*, **23**, 1176.

FOLEY, W. A. & COLE, L. J. (1964) Interaction of Urethan and Fractionated or Regional X-radiation in Mice: Lung Tumor and Leukemia Incidence. *Cancer Res.*, **24**, 1910.

FOLEY, W. A. & COLE, L. J. (1966) Urethan-Induced Lung Tumors in Mice: X-radiation Dose-Dependent Inhibition. *Radiation Res.*, **27**, 87.

HEPPLESTON, A. G. & YOUNG, A. E. (1976) Ultrastructural Changes in the Lung Parenchyma Following Inhalation of Plutonium Dioxide. In *Proc. Path. Soc. Gt. Br. Ir., July* 1976, p. 16.

LEVO, Y., CARNAUD, C., COHEN, I. R. & TRAININ, N. (1974) Increased Incidence of Urethane Induced Lung Adenomata by Autosensitized Lymphocytes. *Br. J. Cancer*, **29**, 312.

LORENZ, E. (1950) Some Biologic Effects of Long Continued Irradiation. *Am. J. Roentgenol.*, **63**, 176.

LORENZ, E., HOLLCROFT, J. W., MILLER, E., CONGDON, C. C. & SCHWEISTHAL, R. (1955) Long-Term Effects of Acute and Chronic Irradiation in Mice. I. Survival and Tumor Incidence Following Chronic Irradiation of 0·11 r per Day. *J. natn. Cancer Inst.*, **15**, 1049.

UPTON, A. C., KIMBALL, A. W., FURTH, J., CHRISTENBERRY, K. W. & BENEDICT, W. H. (1960) Some Delayed Effects of Atom-bomb Radiations in Mice. *Cancer Res.*, **20**, 1.